# Neurological development and iron supplementation in healthy late-preterm neonates: a randomized double-blind controlled trial

**DOI:** 10.1007/s00431-021-04181-1

**Published:** 2021-07-21

**Authors:** R. Luciano, D. M. Romeo, G. Mancini, S. Sivo, C. Dolci, C. Velli, A. Turriziani Colonna, G. Vento, C. Romagnoli, E. M. Mercuri

**Affiliations:** 1 Neonatology Unit, Department of Woman and Child Health and Public Health, Child Health Area, Fondazione Policlinico Universitario A. Gemelli, IRCCS, Catholic University of Sacred Heart, Rome, Italy; 2 Pediatric Neurology Unit, Fondazione Policlinico A. Gemelli IRCCS, Rome, Italy; 3 Department of Woman and Child Health and Public Health, Child Health Area, Catholic University of Sacred Heart, Rome, Italy; 4 Neonatology Unit, Department of Woman and Child Health and Public Health, Child Health Area, Fondazione Policlinico Universitario A. Gemelli, IRCCS, Rome, Italy; 5 Pediatric Neurology Unit, Department of Woman and Child Health and Public Health, Child Health Area, Fondazione Policlinico Universitario A. Gemelli, IRCCS, Catholic University of Sacred Heart, Rome, Italy

**Keywords:** Late preterm, Psychomotor development, Iron deficiency, Iron supplementation

## Abstract

Late-preterm infants (LPT) are at increased risk for long-term neurodevelopmental sequelae and iron deficiency. The aim of the study is to assess the positive effect of iron supplementation on psychomotor development in healthy LPT. We designed a randomized placebo-controlled double-blind trial dividing the newborns into two groups. Every patient was assessed using the Griffiths Mental Development Scales (GMDS)-II edition at 12-month post-conceptional age. The study was performed at the Neonatology Unit of our Hospital, in Italy. Sixty-six healthy LPT infants born between 34^0⁄7^ and 36^6⁄7^ weeks of gestational age were enrolled in the study. One group received martial prophylaxis from the third week of life to 6 months of post-conceptional age (2 mg/kg/day of iron pidolate), the other received placebo. Fifty-two of the enrolled infants were assessed using the GMDS at 12-month of post-conceptional age. Statistical analysis of the mean scores of the Griffiths subscales was performed. There was a difference in the mean developmental quotient (DQ) (p < 0.01) between the two groups: iron group mean DQ 121.45 ± 10.53 vs placebo group mean DQ 113.25 ± 9.70. Moreover, mean scores of the Griffiths subscales A, B, and D showed significant differences between the two groups (scale A p < 0.05, scale B p < 0.02, scale D p < 0.01, respectively).

*Conclusions:* We recommend that all LPT neonates receive iron supplementation during the first 6 months of life in order to improve their 1-year neurodevelopmental quotient.

**Table:** 

**What is Known:** *• Late-preterm infants (LPT) are at increased risk for long-term neurodevelopmental sequelae and also for iron deficiency.* *• Iron deficiency is an independent risk factor for adverse neurological outcomes.*	
**What is New:** *• Healthy late-preterm who received iron supplementation during the first 6 months of life achieved better neurological outcomes at 12-month post-conceptional age than LPT who received placebo.* *• Our study strongly supports the need for the implementation of martial prophylaxis in LPT neonates.*

**What is Known:**

*• Late-preterm infants (LPT) are at increased risk for long-term neurodevelopmental sequelae and also for iron deficiency.*

*• Iron deficiency is an independent risk factor for adverse neurological outcomes.*

**What is New:**

*• Healthy late-preterm who received iron supplementation during the first 6 months of life achieved better neurological outcomes at 12-month post-conceptional age than LPT who received placebo.*

*• Our study strongly supports the need for the implementation of martial prophylaxis in LPT neonates.*

## Introduction

The term “late-preterm infants” (LPT) is used to define infants born at 34^0⁄7^ through 36^6⁄7^ weeks of gestation. They account for more than 70% of all preterm births [1].

Several papers have reported that LPT infants are at increased risk for neonatal morbidities and long-term neurodevelopmental sequelae, when compared to infants born at term [2–7]. It has also been reported that LPT newborns are at risk of developing iron deficiency (ID), due to both limited reserves and increased iron requirements [8–11]. As extensively demonstrated by preclinical studies in rodents [12–16] and human trials [6, 17], iron deficiency is an independent risk factor for adverse neurological outcomes.

Despite the recommendations of the American Academy of Pediatrics (AAP) and the European Society for Pediatric Gastroenterology Hepatology and Nutrition (ESPGHAN) Committee [18, 19], iron supplementation, as shown in surveys from the USA and Italy [20, 21], is not a widespread clinical practice in LPT as it is in neonates born at lower gestational ages.

The aim of our study is to assess the effects of iron supplementation on neurological development in healthy LPT infants evaluated at 12-month post-conceptional age.

## Patients and methods

### Study design

We designed a randomized placebo-controlled double-blind trial (RCT) in order to assess the possible effect of iron supplementation on psychomotor development at 1-year post-conceptional age (primary outcome of the study) in healthy LPT. The RCT was sponsored by Pediatrica Specialist®, that provided both iron and placebo medications.

LPT neonates born between the 1st of January 2017 and the 31st of December 2017 were assessed for eligibility.

### Selection criteria

The study population includes healthy LPT neonates admitted to the rooming-in ward. LPT neonates were not considered for eligibility in case of admission to the intermediate neonatal care unit or intensive care unit.

Neonates were excluded from enrollment in case of the following:

- BW less than 2000 g, severe intrauterine growth restriction or small for gestational age (< 3° centile),
- major congenital anomalies, suspected syndromes, or congenital infections
- neonatal asphyxia or respiratory distress
- neurological and/or neurosensory disorders, cerebral ultrasound anomalies, or hematologic disorders
- uncertain gestational age (GA)
- neonates not legally recognized by parents.

GA was evaluated according to the first-trimester ultrasound scans or, when not available, with the last menstrual period confirmed by Ballard’s score [22]. All eligible neonates were submitted to cranial ultrasound scan (CUS) assessment through the anterior fontanelle at birth to exclude the presence of major cerebral lesions. CUS was always performed or supervised by the same operator (RL). A HP Hewlett Packard Infinity Point equipped with a 5–7.5 Hz probe was used for CUS investigation.

### Study population

**Fig. 1:**
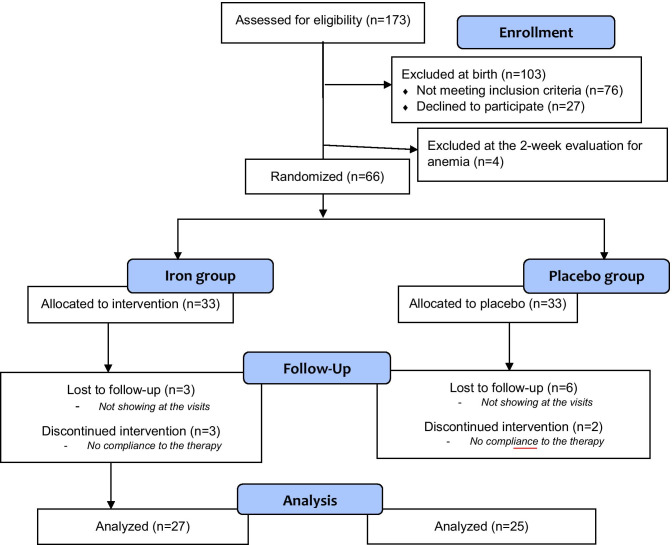
Study population (CONSORT flow diagram)

During the study period, 173 neonates were assessed for eligibility when admitted to the Nursery, shortly after birth. They stayed in the Nursery for a 6-h clinical observation, before being admitted to the rooming-in ward. According with the exclusion criteria, 32 children were excluded for uncertain GA, 3 were not recognized by parents, 7 presented with asphyxia at birth and 3 infants developed respiratory distress, 4 had congenital infections, 16 had congenital malformations or syndromes, 7 patients were excluded because of cerebral anomalies at CUS, and 4 were excluded because of sepsis suspicion. The parents of 27 eligible LPT infants did not consent to the study. A number of 70 healthy LPT infants were enrolled in the study by a neonatologist. At 2 weeks of life, they were submitted to red blood count evaluation to exclude anemia before randomization. Four neonates were anemic; they were excluded from the randomization and started iron treatment. A number of 66 neonates were the final study population that was randomized in two equal groups: 33 patients in the iron group and 33 patients in the placebo group (Fig. 1). Each neonate received a sequentially numbered white not labeled container. Iron and placebo containers were collected, identified as iron or placebo, sequentially numbered, and randomized in the pharmacy of our institution.

Informed consent was obtained from the parents by a neonatologist in the rooming-in ward before discharge.

### Intervention

Iron supplementation consisted in iron pidolate in drops (PediaFer Plus® drops 15 mL) at a dosage of 2 mg/kg/day orally in two administrations each day. At enrollment, parents received a schedule reporting how they should increase the amount of drops to be given as infant’s weight increased.

The iron group received martial prophylaxis starting at 14 days of life until 6 months of age. The placebo group received placebo, similar in shape and flavor to the iron pidolate (PediaFer Plus®) provided to the iron group, and it was administered for the same time interval.

Children who had not assumed the study medication more than 80% of the assigned days were defined as poor compliers and excluded from the study at the 6-month evaluation.

### Clinical and laboratory assessment

All enrolled neonates had a complete blood count check at two weeks of life, before entering the randomization, to exclude anemia. Cutoff for anemia at 2 weeks of life was hemoglobin 11 g/dL for 35–36 GA infants and 10.5 g/dL for 34 GA infants according to Jopling et al. reference values [23]. Four patients were excluded from randomization at this step because of anemia. A general clinical examination was performed by a pediatrician at the age of 6 months and at the post-conceptional age of 12 months to ascertain the compliance in treatment regimen, to collect clinical history and duration of breastfeeding since discharge, to assess infants’ growth and general wellness, and to exclude the presence of anemia with a red blood count. Cutoff for anemia at the 6- and 12-month evaluations was hemoglobin 10.5 g/dL [18].

The clinical assessment performed at 12-month post-conceptional age included a psychomotor assessment by child neurologists (DMR, SS). Every patient was assessed using the Griffiths Mental Development Scales (GMDS)-II edition [24]. The GMDS includes five subscales: A (locomotor), B (personal and social development), C (hearing and speech), D (hand and eye coordination), and E (performance). Each scale provides a mental age and a developmental quotient (DQ). The total DQ was calculated from the mean of the developmental quotients obtained in each of the five subscales.

The DQ was considered “normal” if greater than 85, “borderline” between 85 and 70, and suggestive of development delay if less than 70.

All the clinicians (neonatologists and neurologists) involved in the study were blinded to the patients’ treatment.

### Ethical committee approval

The RCT was approved by the Ethical Committee of our institution, Protocol N° 11,218/13. The full trial protocol is accessible at the Archive of the Ethical Committee of our Institution.

### Statistical analysis

We calculated the sample size on the hypothesis that iron supplementation would be able to increase neurodevelopmental GMDS scores. We decided to fix a 7-point DQ improvement in supplemented group looking at the study by Morag et al. [25]. The authors compared the GMDS scores obtained in 124 LPT and 33 term infants at 1 year of age and found significantly lower scores in the preterm group in all subscales. The greatest difference was found in the performance scale being the mean developmental quotient for performance subscale 84 (SD 10) and the expected one 91 (SD 10), resulting in 7-point difference. Setting a significance level of 0.05 and a power of 0.80, the calculated sample size required was determined to be 33 for each study group.

Statistical analysis was performed using GraphPad software 2018®. The Student’s t test for independent data was used to evaluate differences in neurological assessment between the two groups of our study. For all analyses, a p-value < 0.05 was considered significant.

## Results

The compliance with the intervention was verified with parents at the 6-month visit: five neonates did not comply with the intervention protocol because they did not tolerate iron (3 patients with abdominal pain) or placebo (2 patients with regurgitation) assumption, and they were excluded from the study. Nine neonates were lost to follow-up before the 12-month neurologic evaluation.

Fifty-two of the initially enrolled neonates (twenty-seven in the iron group and twenty-five in the placebo group) were assessed using the GMDS at 12-month post-conceptional age (Fig. 1). The two groups had similar baseline clinical features (Table 1). The infants were homogeneous for the most important clinical characteristics: sex, gestational age, APGAR index at 1**′** and 5**′,** and birth weight (non-significant p-value in the analysis between the two groups). A similar distribution between the two study groups was also observed in maternal age, social status, maternal iron status, gestational and type 1 diabetes, preeclampsia, and gestational hypertension. No mother smoked during pregnancy.

**Table 1:** Study population and maternal characteristics

	IRON group (*n* = 27)	Placebo group (*n* = 25)
Sex (M)	13/27	15/25
GA (weeks)	35.33 ± 0.73	35.2 ± 0.76
Birth weight (g)	2465 ± 410.22	2522.17 ± 392.72
Apgar score 1′	8.48 ± 0.77	8.57 ± 0.93
Apgar score 5′	9.4 ± 0.5	9.67 ± 0.48
Maternal age ± SD	34.74 ± 5.09 years	34.36 ± 5.70 years
Social status		
Working class	8	5
Middle class	13	14
Professional	6	6
Caesarian section	17	13
Maternal iron deficiency without anemia	6	4
Maternal anemia	1	1
Gestational diabetes	3	1
Diabetes type 1	0	1
Preeclampsia	2	4
Gestational hypertension	1	3
Smoke during pregnancy	0	0

Table 2 shows breastfeeding data: 12 out of 27 infants in the iron group and 9 out of 25 infants in the placebo group were breastfed during the first 6 months of life. All the infants assessed were found in good clinical conditions when evaluated at the age of 6 and 12 months. Growth parameters collected at 6- and 12-month evaluations showed a regular growth velocity according to WHO Growth Charts in both groups (Table 2). Four patients were admitted to the Emergency Department for infections: two patients in the placebo group (bronchiolitis and otitis) and two children in the iron group (gastroenteritis caused by rotavirus and urinary tract infection). Blood tests were run at 2 weeks, 6 months, and 12 months, and the mean hemoglobin values were as follows: 14.66 ± 1.86 g/dL at 2 weeks, 11.95 ± 0.83 g/dL at 6 months, 11.9 ± 0.94 g/dL at 12 months in the placebo group, and 14.16 ± 1.72 g/dL, 11.7 ± 1.0 g/dL, and 11.9 ± 0.73 g/dL, respectively, in the iron group (Table 2). Two infants in the placebo group were found anemic at the 12-month blood count evaluation (Hb 9.6 and 9.7 g/dL, respectively), and they were submitted to iron treatment. No infant was found anemic in the iron group.

**Table 2:** Study population: clinical and laboratory data

	Iron group (*n* = 27)	Placebo group (*n* = 25)
Breastfeeding: exclusive or breastfeeding + formula milk (0–6 months)	12	9
Length at 6 months (SDS-WHO charts)	−0.12	−0.55
Length at 12 months (SDS-WHO charts)	0.01	0.06
Weight at 6 months (SDS-WHO charts)	−0.4	−0.2
Weight at 12 months (SDS-WHO charts)	0.18	0.53
Head circumference at 6 months (SDS-WHO charts)	0.38	0.58
Head circumference at 12 months (SDS-WHO charts)	0.61	0.97
Hemoglobin at 14 days	14.16 ± 1.72	14.66 ± 1.86
Hemoglobin at 6 months of age	11.70 ± 1.00	11.95 ± 0.83
Hemoglobin at 12 months post-chronological age	11.9 ± 0.73	11.85 ± 0.94

There was a difference in the mean developmental quotient (DQ) (*p* < 0.01) between the iron group (confidence interval CI 117.29; 125.62) and the placebo group (CI: 109.25; 117.25) (Table 3). Treated infants had higher scores in all Griffiths’ scales.

**Table 3:** Mean developmental quotient of the Griffiths’ subscales in the two groups and SD

	Iron group (*n* = 27)		Placebo group (*n* = 25)		*P* Value
	Mean	SD	Mean	SD	
DQ (A)	127.73	18.30	117.37	15.53	*p* < 0.05
DQ (B)	123.76	11.50	116.29	10.60	*p* < 0.02
DQ (C)	117.90	13.04	112.86	13.33	Non-significant
DQ (D)	120.67	14.25	109.06	14.42	*p* < 0.01
DQ (E)	117.19	15.01	110.66	11.70	Non-significant
Mean DQ	121.45	10.53	113.25	9.70	*p* < 0.01

The analysis of the Griffiths subscale mean scores showed differences in scale A (motor scale) (*p* < 0.05), B (behavior) (*p* < 0.02), and D (hand and eye coordination) (*p* < 0.01). The differences found in the C (hearing and speech) and E (performance) scales did not reach statistical significance (Table 3).

None of the patients recruited in the study had DQ scores suggestive of developmental delay. Three infants in the placebo group had borderline scores compared to none in the iron group.

## Discussion

LPT infants are at risk of neurologic impairments, developmental disabilities, school failure, and behavior and psychiatric problems [26, 27]. Research on the efficacy and benefits of specific therapies in this population could help in reducing impairment in psychomotor development and behavioral problems. We decided to perform a RCT study looking at the possible benefit of martial therapy on the psychomotor outcome at 1 year of post-conceptional age in LPT healthy infants. The importance of iron stores for central nervous system development in the first years of life is widely recognized. The magnitude of iron stores depends on GA at birth. Because iron supply from the placenta is abruptly interrupted with preterm birth, iron storage is lower in LPT than term infants [11]. In contrast to minor storage at birth, a higher supply of iron is required during the first months of life after a preterm birth because growth velocity is maximally increased between 28 and 38 weeks of gestation [9]. The iron stores of preterm infants may, consequently, be depleted much earlier than 5–6 months of life, when iron-rich complementary foods are added to the diet. Thus, iron supplementation is needed in preterm infants to meet the sustained high demands for hematopoiesis, tissue accretion, and brain development [10, 26]. Iron is important for neurological development due to its determinant role in dendritic arborization, myelination, neurotransmitters’ metabolism, glucose homeostasis, and metabolites utilization in the hippocampus [12, 13, 27]. Several studies have also shown the negative effects of iron deficiency in cognitive and motor functions, socio-emotional behavior, auditory and visual function [6, 17]. The American Academy of Pediatrics [19] recommended that all preterm infants should have an iron intake of at least 2 mg/kg per day through 12 months of age, which is the amount of iron supplied by iron-fortified formulas, and that preterm infants fed human milk should receive an iron supplement of 2 mg/kg per day, by 1 month of age. This regimen should be continued until the infant is weaned to iron-fortified formula or begins eating complementary foods that supply 2 mg/kg of iron per day. The ESPGHAN Committee on Nutrition recommended iron supplementation for preterm infants with BW < 1800 g, extended to MLBW infants (BW 2000–2500 g) in the 2014 position paper, for a 6-month period, not mentioning whether formula fed infants should be supplemented [18]. There is no consensus on the amount of iron to be supplemented, the length of treatment, and the opportunity to treat formula fed LPT infants. An American study from the USA showed that, among preterm breastfed and mixed-fed infants, none received oral iron supplements 3 times per week before 3 months of age, 2% received them at 3 months, and 13% received them at 10.5 months [21]. One Italian study concerning iron prophylaxis in Piemonte, Marche, and Lazio [20] found that iron for MLBW is recommended in the 25% of ICU and in the 21% of 1st–2nd level centers, and that iron for LPT is recommended in the 26% of ICU and in the 7% of 1st–2nd level centers. Thus, iron supplementation is not a standard policy in this population of neonates. Our study was designed to support the importance of iron supplementation for psychomotor development both in breastfed and in formula fed LPT infants. We excluded from the study neonates with BW less than 2000 g because we considered not ethic giving a placebo in this BW group.

Some infants may not tolerate the iron assumption as shown in our study. Better tolerance to iron treatment may be achieved in the clinical practice testing the individual response to the different available iron medications or attempting to reintroduce the iron treatment when the baby is older (late iron prophylaxis starting after 4 weeks of life). The importance for iron prophylaxis is further underlined by the finding of two anemic infants in the placebo group and none in the iron group at the 12-month evaluation. The study was not designed to analyze the effect of iron prophylaxis on iron deficiency anemia. Improved iron status and reduction in iron deficiency and anemia were already shown in previous reviews [28, 29]. Our study shows no difference in growth parameters and prevalence of infections between the two groups throughout the first year of life according with the conclusions of a recent review that was looking at the effect of iron prophylaxis in preterm and low-birth-weight infants [29]. No association between iron supplementation and adverse clinical outcomes was found by the Authors. High-quality evidence regarding the long-term effects of iron supplementation in terms of growth and general health as well as risk of iron overload are still lacking and could be the object of future investigations.

Analyzing the whole recruited population, we found three scores indicative of “borderline” psychomotor development. They were all found in the placebo group, while none of the infants in the iron group presented borderline or pathological scores. The results in the placebo group are consistent with the natural history of LPT infants [3, 4]. These results should not be driven by possible bias in the selection of the groups as they had similar clinical variables. LPT infants show a global immature neuromotor development in specific neurological items (tone, posture, movements, and reflexes) compared to full-term infants, following a specific developmental trend during the first years of age. No differences are described in the hearing and speech subscale, and this was confirmed in our sample. The neuromotor fall applies in LPT probably due to a brain immaturity and an increased vulnerability to injury, as the last 6 weeks of gestation are essential for the cortical gray and white matter development [30]. Iron supplementation could have improved the brain development in LPT mainly in the neurological areas that are usually delayed in these infants as showed by the higher scores in infants in the iron group than those in the placebo group; this could further justify the higher DQ in the iron group.

Berglund et al. reported a lower rate of behavioral problems on the Child Behavior Checklist (CBCL) at 3.5 and 7 years in moderately low birth weight infants (MLBW = BW 2000–2500 g) supplemented with iron when compared to a placebo group. They demonstrated a more favorable outcome in supplemented versus not supplemented children when they were tested with the Child Behavior Checklist, while they did not find any significant group differences in cognitive scores [31, 32]. The population study in Berglund et al. paper was selected on the basis of birth weight independently on GA, thus including LPT as well as term infants. Previous studies analyzing the effect of iron supplementation on neurologic and psychomotor development were dedicated to very-low-birth-weight infants (BW < 1500 g), population not comparable to LPT in terms of perinatal clinical complications, and late neurodevelopmental outcome [28, 29]. To our knowledge, our RCT study is the first one that analyzes the effect of iron prophylaxis on psychomotor development selectively focusing on LPT population.

Limitations of the study are the following: (1) five neonates did not comply with the medication assumption and were excluded from the study according to the protocol approved by the Ethical Committee, and 9 patients were lost to follow-up before the twelve-month neurodevelopmental evaluation. (2) Neurodevelopmental assessment at 12-month age does not provide the same predictive value as evaluations in pre-school and school age and could overestimate the real developmental quotient. Ten percent of the infants assessed in our study reported very high scores. This was mainly observed in the iron group and may have had an effect in the great differences between the two groups. The data obtained in the Griffiths scales should be read as general development indicators and should be fortified by a long-term follow-up. Our data, obtained in a relatively small cohort, provide preliminary information that could be used to power larger studies that would allow a better stratification by sex, gestational age, breast milk assumption, and other variables.

## Conclusions

Our data show that LPT neonates who received iron supplementation during the first 6 months of life achieved significantly better neurological outcomes at 12-month GMDS assessment than placebo group in terms of total DQ and A, B, and D subscales. Therefore, our study strongly supports the need for implementation of martial prophylaxis in this population.

A limit of the study is the age at neurodevelopmental assessment, which has a less predictive value than preschool or school age evaluations. Reevaluation of children at pre-scholar age would be of interest as well as collecting data on larger populations with the aim to perform assessments for subgroups based on sex, gestational age, and breast milk assumption.

## Data Availability

Data are available at Fondazione Policlinico Gemelli IRCCS.

## Abbreviations

AAP: American Academy of Pediatrics
BW: Birth weight
CI: Confidence interval
CUS: Cranial ultrasound
DQ: Developmental quotient
ESPGHAN: European Society for Pediatric Gastroenterology Hepatology and Nutrition
GA: Gestational age
GMDS: Griffiths mental development scales
ID: Iron deficiency
LPT: Late-preterm
MLBW: Moderately low birth weight
RCT: Randomized controlled trial
SD: Standard deviation
